# Barriers and facilitators to uptake of the school-based HPV vaccination programme in an ethnically diverse group of young women

**DOI:** 10.1093/pubmed/fdv073

**Published:** 2016-10-17

**Authors:** Harriet Batista Ferrer, Caroline L. Trotter, Matthew Hickman, Suzanne Audrey

**Affiliations:** 1 School of Social and Community Medicine, University of Bristol, Bristol BS8 2PS, UK; 2 Department of Veterinary Medicine, University of Cambridge, Cambridge CB3 0ES, UK

**Keywords:** adolescents, decision-making, ethnicity, health inequalities, HPV vaccine

## Abstract

**Background:**

To identify the barriers and facilitators to uptake of the HPV vaccine in an ethnically diverse group of young women in the south west of England.

**Methods:**

Three school-based vaccination sessions were observed. Twenty-three young women aged 12 to 13 years, and six key informants, were interviewed between October 2012 and July 2013. Data were analysed using thematic analysis and the Framework method for data management.

**Results:**

The priority given to preventing cervical cancer in this age group influenced whether young women received the HPV vaccine. Access could be affected by differing levels of commitment by school staff, school nurses, parents and young women to ensure parental consent forms were returned. Beliefs and values, particularly relevant to minority ethnic groups, in relation to adolescent sexual activity may affect uptake. Literacy and language difficulties undermine informed consent and may prevent vaccination.

**Conclusions:**

The school-based HPV vaccination programme successfully reaches the majority of young women. However, responsibility for key aspects remain unresolved which can affect delivery and prevent uptake for some groups. A multi-faceted approach, targeting appropriate levels of the socio-ecological model, is required to address procedures for consent and cultural and literacy barriers faced by minority ethnic groups, increase uptake and reduce inequalities.

## Background

In the UK, the human papillomavirus (HPV) vaccine is routinely available for young women aged 12 to 13 years^1^ to reduce cervical cancer incidence and mortality. Equitable uptake of UK HPV vaccination programmes has been demonstrated in relation to deprivation indices.^2–4^ This is encouraging given that socioeconomically disadvantaged women are more likely to develop cervical cancer^5^ yet less likely to attend for screening.^6,7^ However, in the south west of England, the odds ratio of HPV vaccination initiation in young women belonging to minority ethnic groups is approximately half in comparison to White British young women.^4^ Lower uptake has also been shown in other UK settings.^3,8^ This is of concern as Asian and Black women aged over 65 years may be at increased risk of developing cervical cancer,^9^ but less likely to attend for cervical cancer screening.^10,11^

There is a danger that existing cervical cancer inequalities could widen if uptake of the HPV vaccination programme is lower among populations already at greater risk. Therefore, the aims of this study were to identify the barriers and facilitators to uptake in an ethnically diverse group of young women, with previously identified lower uptake,^4^ and to make recommendations to increase uptake.

## Methods

### Sampling and recruitment

Data collection took place in the south west of England between October 2012 and July 2013. Three state-funded, comprehensive schools were purposively selected on the basis of HPV vaccine initiation (range: 65–84%), and the proportion of the student population defined as belonging to a ‘non-White British’ ethnic group, from this point forward referred to as minority ethnic group (range: 20–74%) (unpublished routinely collected data). Multiple perspectives were sought for this study to gain a more comprehensive understanding of factors affecting uptake. The lead school nurse and a key staff member at each school were given information about the study and invited to participate in an interview.

A two-tiered system of consent was used to recruit young women. In the study schools, the parents of all young women eligible for vaccination according to the English national immunization schedule were sent an information pack with a reply slip to be completed if they did not wish their daughter to take part (parental opt-out). Young women whose parents had not opted them out were given an information leaflet and asked for their assent to complete a short questionnaire providing their basic details (including ethnicity and Free School Meal entitlement) and vaccination status. This two-tier consent procedure for low-risk research studies with young people has been shown to result in higher recruitment rates, especially those from socioeconomically disadvantaged backgrounds.^12,13^

A sampling frame, stratified by vaccination status and ethnicity (Black/Black British, Asian/British Asian, White British and Other/Mixed), was created, and potential participants were then randomly selected from each strata using a computer-generated number. Selected young women were able to nominate a peer of their choice to participate in the interview with them if they wished. The views of young women from minority ethnic groups and White British young women were sought to gain understanding of factors affecting uptake unrelated to ethnicity or culture. The young women were given an information sheet and invited by the study researcher (H.B.-F.) to participate, for which written parental consent was sought.

Participants received a £10 gift voucher to reimburse them for their time. Ethical approval was obtained from the Faculty of Medicine and Dentistry Committee for Research Ethics, University of Bristol.

### Data collection

In each school, a vaccination session was observed during which detailed field notes about the context and any specific incidents relevant to uptake recorded. Young women were interviewed alone or with a peer. All key informant interviews took place alone. The interviews took place either in the school, home or place of work of the participant. Semi-structured topic guides, informed by the findings of a previous qualitative synthesis,^14^ were used and covered vaccination beliefs, experiences of the HPV vaccination programme, decision-making and consent, and cultural and religious beliefs.

Interviews were carried out until saturation was achieved and no new issues arose*.* To minimize researcher bias, the interviewer (H.B.-F.) was careful to remain neutral with respect to her personal views and to the responses provided. All interviews were digitally recorded with the permission of the participant and confidentiality maintained.

### Data analysis

As data collection progressed, interview recordings were transcribed verbatim, double checked and potentially identifying information removed. Contextual notes were recorded in the transcripts.

The analysis was based on methods from thematic analysis^15^ and the Framework approach to data management.^16,17^ Familiarization with the data began by reading and re-reading the transcripts. Sections of text were coded, with multiple codes being allocated where appropriate. Coding was simultaneously inductive (emerging form the data in the transcripts) and deductive (based on the research questions and constructs previously identified).^14^ Similar codes were grouped together to create a thematic framework comprising a hierarchy of themes and sub-themes. Codes were double checked by the same researcher to ensure consistency and accuracy. Analysis was undertaken independently by one researcher (H.B.-F.) with discussions held with a study author (S.A.) as analysis progressed.

Separate charts were constructed around key themes for young women (organized by vaccination status) and key informants using the Framework Matrix within QSR NVivo10 software. Streamlined versions of the charts were produced as the process of summarizing the data progressed. In these charts, key terms and phrases were retained while repetition within studies and extraneous text were removed.

## Results

### Participants

Key informant interviews (range: 24–46 min) took place with three school staff and three school nurses. Of 34 young women invited for interview (either purposively selected or nominated by their peer), parental consent was obtained for 23 young women who were all subsequently interviewed in 17 paired or unpaired interviews (range: 9–33 min). Reasons for non-participation included parental refusal [1], self-refusal [1] and unreturned parental consent form [9]. Seventeen young women were from a minority ethnic group. The majority of young women interviewed had received the vaccine [17]; most received the vaccine in the school setting [14]. Seven young women were unvaccinated at the time of the interview (Table 1).

**Table 1:** Characteristics of young women who participated in an interview

*Characteristic*	**n* (%)*
Vaccination status	
Vaccinated	16 (69.6)
Not vaccinated	7 (30.4)
Vaccination setting	
School	13 (81.3)
General practice	3 (18.7)
Ethnicity	
White British	6 (26.1)
Black	10 (43.5)
British Asian	2 (8.7)
Chinese or other	5 (21.8)
Free school meal entitlement	
Yes	12 (52.2)
No	9 (39.1)
Unknown	2 (8.7)
Religion	
Muslim	10 (43.5)
Christian	5 (21.8)
None	7 (30.4)
Unknown	1 (4.3)
First language English	
Yes	8 (34.8)
No	13 (56.5)
Unknown	2 (8.7)
Length of interview (min)	9–33

### Themes

The sections below provide a summary of the key issues (vaccine beliefs, priority, sexual mores, information needs, and decision-making and consent) identified as influential to uptake. Illustrative quotations were chosen, because they were expressed concisely and typify responses relating to the themes (Tables 2 and 3). Due to potential detrimental changes to the dynamics of the interviews if translators were present, all interviews took place in English. Some participants were not speaking in their first language (*n* = 13), and their words are presented verbatim.

**Table 2:** Key themes: vaccine beliefs, priorities and sexual mores

Vaccine beliefs	‘The female parents were saying to me that actually they're mistrusting...of our NHS service and giving vaccines to children, because when they've been in, some of them have come from war torn areas, and their children have been given vaccines by people like the Red Cross, or UNICEF, or charitable agencies and they're not even sure what their child is being given’ [School nurse 1]
	‘You get people who buy into it, and people who don't, you know. “Oh, it's all mumbo jumbo, I'm never going to give my child a vaccination ever”’ [School staff, School 3]
	‘Yeah I've had lots of injections ‘cos of the tropical diseases and stuff’ [ID229, vaccinated, British Somali]
	‘Most of them were scared about the side effects, but I wasn't really too worried about that’ [ID345, vaccinated, Black/Black British]
	‘It's too scary for me. I cried, but as a child when I used to get my injections, I used to cry, I used to bite my hand, I couldn't stand needles’ [ID340, unvaccinated, Black/Black British]
Priority	‘The students that are away are contacted by myself to make sure that they wanted the injection. If they did…I'll ring their parents, they have to contact their GP’ [School staff, School 2]
	‘In this setting, who has the time and capacity to follow up for a letter for a vaccination? If they really wannit they will bring their letters in … You've got ones which are just, like, “I don't really care,” you get a total spectrum, parents that are interested in their kids and parents that aren't’ [School staff, School 3]
	‘It can help them in the future so they don't get cancer’ [ID312, unvaccinated, White British]
	‘I just think that it's better to have the vaccine than not have it, like, at least with the vaccine you've got a chance, like, to slightly lower the risks, whereas without the vaccine you don't really know’ [ID337, vaccinated, Black/Black British]
	‘She [mother] said it was a good thing to have after her [cervical cancer] scare’ [ID211, vaccinated, White British]
	‘You've got ones which are just, like, “I don't really care,” you get a total spectrum, parents that are interested in their kids and parents that aren't’ [School staff, School 3]
Sexual mores	‘We see lots of children that are sexually active at 13…I would say in some areas that we're working, it's the best thing to do really, it's the best time’ [School nurse 3]
	‘Some people will say that because they fear it will encourage their young girls to be sexually active, and they endorse marriage and sex after marriage…they really don't want this influence on their child’ [School nurse 1]
	‘I never really wanted them [HPV vaccination injections] and my mum was just, like, if you're not sexually active then you obviously won't need it until later on’ [ID345, unvaccinated, Black/Black British]
	‘She [mother] said that I didn't really need it now because, even if I do need it and want it done, I can just go to the doctors’ [ID312, unvaccinated, White British]

**Table 3:** Key themes: information needs, decision-making and consent

Information needs	‘Here there are 38 languages…so what parents are very good at doing here is they'll get a letter in English and they'll find a friend to interpret it for them, which I don't think is good enough…there needs to be more translations into general languages’ [School staff, School 2]
	‘The consent forms are signed on both sides which gives you the impression that they don't understand the form…They've signed to say they want the vaccine and signed that they don't want the vaccine’ [School nurse 1]
	‘She [mother] didn't want me to do it because she didn't know anything about this thing, and it said, when she investigated, first she went to ask the lady and then she went to the GP, and then when they said yes she was a bit more comfortable about the vaccine’ [ID255, vaccinated, Turkish]
	‘My mum doesn't speak very good English so I was explaining it to her’ [ID202, vaccinated, Black/British Black]
	‘I didn't like the idea at first but then once I read about it, and then I was, like, I want to have the jab now ‘cos I know about it’ [ID230, vaccinated, White British]
	‘Well they gave us like a bunch of papers, but then, personally I didn't really read it…It was so long! Yeah, and I didn't really care’ [ID340, unvaccinated, Black/Black British]
Decision-making and consent	‘My mum found out by me going to my mum and saying “I missed the letter for the HPV vaccine, can you, like, ring up school and tell them that it's ok for me to have it?” But she kept forgetting and then we eventually called the doctors, so I got it there’ [ID211, vaccinated, White British]
	‘They let me choose, my parents…they didn't force me to take it, and I said I didn't want to take it’ [ID340, unvaccinated, Black/Black British]
	‘I think if the child really wants it, they have to persuade their mum’ [ID243, vaccinated, Asian/British Asian]
	‘We obviously send out the information pack with the consent form in, and most of those just come back straight forward, obviously you always get the ones that don't, and then we can send out a reminder text’ [School staff, School 1]
	‘Some of the parents don't even see their children after school, they're in bed, they write their own little notes in, and they might just squiggle their signature on the form, so maybe there's some that don't even know they're having it’ [School staff, School 1]
	‘Unless we've got parental consent, or which we hope that that's the parent's signature that is on there, we don't do Fraser competence, we say that they have to go to their GP surgery’ [School nurse 2]
	‘There is no way you can be giving a vaccination to a child without their parents’ consent. That is beyond crazy!’ [School staff, School 3]
	‘They kept sending me one every day, like, in the post, so I keep throwing it in the bin, I'm like, don't wannit’ [ID340, unvaccinated, Black/Black British]

### Vaccine beliefs

Key stakeholders suggested that some parents were wary of vaccinations or have beliefs that oppose vaccination. However, although unable to provide details, most of the young women interviewed were aware that they had received childhood or travel vaccines. Some young women indicated implicit trust in the advice of healthcare professionals to receive vaccinations. Nevertheless, fear of vaccination, especially needles, and side-effects were important reasons why some young women resisted vaccination (Table 2).

### Priority

Key informants indicated that prevention of cervical cancer was an important reason to prioritize young women's receipt of the vaccine. However, differing levels of school staff commitment and ability to prioritize the HPV vaccination programme were apparent with implications for uptake. Family experiences of different types of cancer, inability to predict the future development of illness and anticipated regret encouraged young women. When vaccination was missed in the school setting, uptake depended on the ability of individuals to prioritize and arrange an appropriate GP appointment. Although some unvaccinated young women felt the HPV vaccine was important, key stakeholders and young women reported that indifference by families could act as a barrier (Table 2).

### Sexual mores

Key informants agreed that the recommended age to vaccinate was appropriate to ensure young women are adequately protected prior to sexual debut. However, the stigma of vaccination against a sexually transmitted infection was perceived by key informants to prevent vaccination uptake for some young women within cultures advocating monogamy and prohibiting sexual contact outside of marriage. In contrast, most young women did not refer to the link between the HPV vaccine and sexual behaviour. However, three unvaccinated young women from minority ethnic groups did connect the HPV vaccine with sexual activity which appeared to reduce perceptions of need. The relatively young age for vaccination was also considered a pertinent issue for some families not belonging to minority ethnic groups (Table 2).

### Information needs

Some young women commented that their parents had sought additional information, through the internet or healthcare professionals. Lack of understanding and literacy issues, not limited to minority ethnic families, could present difficulties. The importance of information in multiple languages, and provision of verbal information, to families was highlighted. However, improving dialogue with families who were more generally perceived as disengaged, and less likely to return consent forms for a range of school-based activities, was considered problematic.

Almost all young women reported receiving information about the vaccine through the school, delivered either in an assembly or a lesson. However, two unvaccinated young women said they had not heard about the HPV vaccine in the school setting. Despite apparently low levels of knowledge, most young women felt that they were informed. Some mentioned that information provision had encouraged them to receive the HPV vaccine. The importance of providing age appropriate information to engage and inform young women was discussed (Table 3).

### Decision-making and consent

The majority of vaccinated young women indicated that decisions were made by their parents, or with other adults, irrespective of their own perspective. However, the accounts of two young women implied that they had been instrumental in ensuring that they had received the HPV vaccine after missing vaccination in the school setting. Unvaccinated young women also claimed to have some autonomy in decision-making with parents supporting their decision. Some young women felt their peers should be able to exercise autonomy and refuse vaccination. However, key informants and some young women judged the level of maturity of vaccine eligible young women inhibited decision-making.

Varying levels of commitment and capacity by the schools to ensure consent forms were returned were evident. Chaotic family life, as opposed to active opposition to the HPV vaccine, was offered as an explanation by school nurses for failure to return the consent forms. Where parents were disengaged with the consent procedure, key stakeholders suggested that young women may take on greater responsibility for ensuring that they received the HPV vaccine. Some young women perceived they had a role to encourage their parents to provide consent.

Despite the legal rights of young women to be vaccinated without parental consent,^18^ the school nurses annual vaccination training inhibited vaccination without written parental consent, and school nurses and staff were unwilling to be accountable. Relying on young women, who may be ambivalent about the HPV vaccine, to facilitate the consent procedure may also prevent, or delay, uptake. However, even in settings where information was posted directly to parents, young women may still intercept the consent forms if they did not want to receive the vaccine (Table 3).

## Discussion

### Main findings of the study

This study shows that vaccine beliefs and the priority given to preventing cervical cancer influenced young women's HPV vaccine receipt in a universal schools-based programme. Uptake was influenced by differing levels of commitment for the parental consent forms to be returned. Beliefs and values of adolescent sexual activity, particularly relevant to minority ethnic groups, may negatively influence uptake. Lack of accessible information may also act as a barrier.

### Pathways to HPV vaccination

**Fig. 1:**
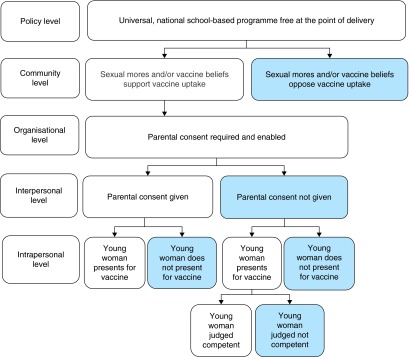
Pathway of HPV vaccine receipt for young women in the south west of England.

The socio-ecological model^21,22^ can be used to illustrate a pathway to HPV vaccine receipt for young women in the UK school-based programme (Fig. 1). This shows that social norms and vaccine beliefs at the community level may influence whether young women receive the HPV vaccine. At the organizational level, the requirement for parental consent is accompanied by varying degrees of commitment from school staff and nurses when seeking consent from more ‘hard to reach’ groups. Some young women contributed to decision-making about the vaccine and exercised some autonomy by persuading their parents to take them to the GP if they missed a session, while others avoided being vaccinated by throwing away information or consent forms. These issues may also be relevant to the recently introduced MenC and Td/IPV school-based vaccination programmes in the UK.

Currently, National Institute for Health and Clinical Excellence guidelines^25^ for reducing inequalities of immunization uptake among children and young people does not specifically address the HPV vaccination programme. Below, we use the socio-ecological model to consider the level at which intervention is required to increase uptake among under-represented groups.

### Policy level

The provision of the HPV vaccine in the UK within the school setting free at the point of delivery has overcome financial and access barriers identified in other settings.^14^ However, consent procedures for young women to be vaccinated without parental consent^18^ were not being followed in the school setting. NHS HPV vaccination programme information states that young women can legally override their parent's decision if they are considered capable of decision-making.^26^ However, in the latest healthcare professional guidance, consenting young women for vaccination without parental consent is not fully addressed.^27^ Clarification is required and could be achieved through issue of policy guidance.

### Community level

Cultural values prohibiting sexual contact outside of marriage appeared to prevent some young women from minority ethnic groups receiving the HPV vaccine. Expectations of young women's sexual behaviour, based on their family's values, may differ from their actual future behaviour and consequent risk of exposure to HPV. Young people from minority ethnic groups have described tensions between conforming to the expectations of sexual behaviour from their family and their local community, with the ‘mainstream’ cultural values to which they are exposed.^28,29^ It may be beneficial to focus on the HPV vaccine as a universal health promotion initiative. Sensitivity is required but information could also enforce messages that a young woman may be at risk of acquiring HPV through her partner.

### Organizational- and interpersonal levels

A key issue that prevents uptake is non-returned parental consent forms. To address this, there is a need to foster better communication between schools, school nurses and parents, and to encourage joint working between school nurses and school staff. This could be achieved by assigning a ‘school champion’ in each setting to chase-up unreturned consent forms. Subsequently, school nurses could target families with unreturned consent forms through follow-up telephone calls to check their wishes. There is some evidence that school nurses perceive this approach as successful in reducing health inequalities and increasing uptake.^30,31^

There was evidence for unmet information needs due to language and literacy barriers, with important implications for informed consent. Provision of information verbally or in the preferred language may be beneficial in ensuring that information reaches, and is accessible, to parents and their daughters.

### What is already known about this topic

A number of relevant systematic reviews comprising studies predominantly from the USA exist. Unaddressed information needs may prevent positive decision-making and seriously impede families’ ability to make informed choices about HPV vaccination.^19^ Currently, evidence for the effectiveness of educational interventions, including those which are culturally appropriate, to increase uptake is lacking.^20^

The socio-ecological model^21,22^ has been used previously to illustrate how a young woman's access to the HPV vaccine in high income countries is shaped by the policy context; social norms and values, particularly in relation to sexual activity; the views and actions of healthcare professionals, and parental consent.^14^ Further, although young women are the main participants and beneficiaries, their views are relatively under-represented in the qualitative literature.^14^

Two UK-based qualitative studies examining young women's perspectives of the HPV vaccine were undertaken shortly after programme introduction. Both studies reported that, despite the majority of participants being vaccinated, they had limited knowledge about the HPV vaccine^23,24^ and were fearful of receiving the vaccine.^24^ Misperceptions of need based on sexual activity and concerns about novelty, safety and efficacy were reported by unvaccinated young women.^23^

### What this study adds

This is the first study which has, from the perspectives of different stakeholders and young women from a range of backgrounds, examined the reasons for lower HPV vaccine uptake among minority ethnic groups in the UK schools-based programme. We have shown that socio-cultural factors, in addition to vaccination and health beliefs, can affect whether young women are vaccinated. We also used the socio-ecological model to make recommendations at the appropriate level to address inequalities in uptake.

### Limitations of this study

Despite best efforts, the number of unvaccinated young women recruited to this study was lower than planned. Three young women who were unvaccinated at the time of questionnaire completion had received the HPV vaccine when the interview took place. Other unvaccinated young women did not return parental consent forms and their views could not be explored.

Young women appeared reluctant to discuss the sexual transmission of HPV. Young women from minority ethnic groups may have been less likely to share this information with the interviewer (White British) due to perceptions of lack of shared experiences. The findings presented in relation to sexual mores are predominantly based on the views of key stakeholders. Further research is required to capture the views of parents of unvaccinated daughters.

Interviews with young women took place during the school year they were eligible for the HPV vaccine. Therefore, their views are unlikely to be significantly affected by recall bias. However, interviews were shorter than anticipated with many of the young women unable to speak in-depth about the HPV vaccine. This may result from their age, their limited involvement in decision-making, lack of knowledge or level of importance accorded to the HPV vaccine.

## Conclusions

The school-based HPV vaccination programme reaches the majority of eligible young women. However, unresolved responsibility for key aspects of the programme, as well as social norms and values, can impact on delivery and prevent uptake for some groups of young women. A multi-faceted approach is required to reduce inequalities in uptake.

## Funding

This work was supported by the Centre for the Development and Evaluation of Complex Interventions for Public Health Improvement (DECIPHer), a UKCRC Public Health Research Centre of Excellence. Joint funding (MR/KO232331/1) from the British Heart Foundation, Cancer Research UK, Economic and Social Research Council, Medical Research Council, the Welsh Government and the Wellcome Trust, under the auspices of the UK Clinical Research Collaboration, is gratefully acknowledged. In addition, the study was supported by the NIHR Health Protection Research Unit in Evaluation of Interventions. The views expressed are those of the author(s) and not necessarily those of the NHS, the NIHR, the Department of Health or Public Health England. Support from the Biosocial Society is also gratefully acknowledged.

## Conflict of interest statement

H.B.-F., M.H. and S.A. have no conflicts of interest to declare. C.L.T. received consultancy payment from GSK for a critical review of a health economic model of meningococcal ACWY vaccine.
